# Quadruplex PCR assay for identification of *Corynebacterium pseudotuberculosis* differentiating biovar Ovis and Equi

**DOI:** 10.1186/s12917-017-1210-5

**Published:** 2017-09-25

**Authors:** Sintia Almeida, Elaine M. S. Dorneles, Carlos Diniz, Vinícius Abreu, Cassiana Sousa, Jorianne Alves, Adriana Carneiro, Priscilla Bagano, Sharon Spier, Debmalya Barh, Andrey P. Lage, Henrique Figueiredo, Vasco Azevedo

**Affiliations:** 1 Instituto de Ciências Biológicas, Federal University of Minas Gerais, Belo Horizonte, MG Brazil; 2 Escola de Veterinária, Federal University of Minas Gerais, Belo Horizonte, MG Brazil; 3 Departamento de Medicina Veterinária, Federal University of Lavras, Lavras, MG Brazil; 4 Centro de Energia Nuclear na Agricultura, University of Sao Paulo, Piracicaba, SP Brazil; 5 Instituto de Ciências Biológicas, Federal University of Para, Belém, PA Brazil; 6 Department of Medicine and Epidemiology, UC Davis School of Veterinary Medicine, Davis, CA USA; 7 Centre for Genomics and Applied Gene Technology, Institute of Integrative Omics and Applied Biotechnology (IIOAB), Nonakuri, Purba Medinipur, WB India; 8 Aquacen - National Reference Laboratory for Aquatic Animal Diseases, Federal University of Minas Gerais, Belo Horizonte, MG Brazil

**Keywords:** Caseous lymphadenitis, Diagnosis, Nitrate reductase, Horse, Sheep, Goats

## Abstract

**Background:**

*Corynebacterium pseudotuberculos*is is classified into two biovars, nitrate-negative biovar Ovis which is the etiologic agent of caseous lymphadenitis in small ruminants and nitrate-positive biovar Equi, which causes abscesses and ulcerative lymphangitis in equines. The aim of this study was to develop a quadruplex PCR assay that would allow simultaneous detection and biovar-typing of *C. pseudotuberculosis*.

**Methods:**

In the present study, genomes of *C. pseudotuberculosis* strains were used to identify the genes involved in the nitrate reduction pathway to improve a species identification three-primer multiplex PCR assay. The nitrate reductase gene (*narG*) was included in the PCR assay along with the *16S, rpoB* and *pld* genes to enhance the diagnosis of the multiplex PCR at biovar level.

**Results:**

A novel quadruplex PCR assay for *C. pseudotuberculosis* species and biovar identification was developed. The results of the quadruplex PCR of 348 strains, 346 previously well-characterized clinical isolates of *C. pseudotuberculosis* from different hosts (goats, sheep, horse, cattle, buffalo, llamas and humans), the vaccine strain 1002 and the type strain ATCC 19410^T^, were compared to the results of nitrate reductase identification by biochemical test. The McNemar’s Chi-squared test used to compare the two methods used for *C. pseudotuberculosis* biovar identification showed no significant difference (*P* = 0.75) [95% CI for odds ratio (0.16–6.14)] between the quadruplex PCR and the nitrate biochemical test. Concordant results were observed for 97.13% (338 / 348) of the tested strains and the kappa value was 0.94 [95% CI (0.90–0.98)].

**Conclusions:**

The ability of the quadruplex assay to discriminate between *C. pseudotuberculosis* biovar Ovis and Equi strains enhances its usefulness in the clinical microbiology laboratory.

**Electronic supplementary material:**

The online version of this article (10.1186/s12917-017-1210-5) contains supplementary material, which is available to authorized users.

## Background

*Corynebacterium pseudotuberculosis* is a facultative intracellular bacterium that is the causative agent of caseous lymphadenitis (CLA) in goats and sheep, abscesses and ulcerative lymphangitis in horses and oedematous skin disease (OSD) in Buffalo. It also causes sporadic infections in other species including cattle, camels, llamas, and humans [1–4]. *C. pseudotuberculosis* can be classified in two biovars, based on their ability to convert nitrate to nitrite, nitrate-positive strains are classified as biovar Equi and the nitrate negative ones as biovar Ovis [5]. In sheep and goats, CLA is predominantly caused by biovar Ovis strains, whereas horses and buffalos are mostly infected by biovar Equi strains [6–8]. Infection by *C. pseudotuberculosis* is distributed worldwide, causing significant disease in horse, sheep and goat herds [8, 9]. The main economic losses attributed to *C. pseudotuberculosis* infection include decreased milk production, decreased weight gain, reduced value of hides due to scarring, and the cost of the drugs and labor needed to treat disease [9].

Direct and indirect tests to detect *C. pseudotuberculosis* have already been proposed, such as complement fixation test [10], synergistic hemolysis inhibition test [11], microagglutination assay [12], phospholipase D (PLD) antigen-based ELISA [13] and a multiplex PCR developed by our research group [14]. While these tests are useful for clinical diagnosis in diseased animals, none can differentiate the two biovars of *C. pseudotuberculosis*, which is currently only accomplished by biochemical tests. Differences between biovars are relevant for host and tissue specificity and appear to be associated with virulence [15, 16].

Disease caused by *C. pseudotuberculosis* biovars has different clinical manifestations in the susceptible hosts [6, 8, 17, 18], and biovar identification is important for understanding the epidemiology of infection, and consequently for disease control. Moreover, biovar identification can also have clinical implications. Since cattle can be infected by strains of both biovars, which may have different tissue preferences: biovar Ovis infects chiefly the mammary gland [17] and skin [18] and biovar Equi causes ulcerative lymphangitis and coronet lesions [9, 19, 20].

A dominant genetic characteristic that differentiates the biovars of *C. pseudotuberculosis* is the presence of the nitrate reduction operon in the biovar Equi strains [21]. Additionally, biovar Equi strains have 15 genes that are absent in biovar Ovis strains, including the *narKGHJI* operon, and a gene cluster encoding the molybdopterins *moeB, moaE, molB, molA, moeY, moaC, moeA,* and *moaA* and two hypothetical proteins [21].

Currently, only nitrate reduction test distinguishes *C. pseudotuberculosis* biovars Ovis and Equi [5, 9]. The available phenotypic tests, performed in a laboratory or commercially available, for *C. pseudotuberculosis* identification are usually effective. However, the phenotypic tests can be expensive and unavailable for some laboratories that prefer to use molecular techniques. Currently, clinical microbiology laboratories are experiencing a change from classical to new diagnostic tools as PCR, real-time PCR, sequencing and MALDI-TOF mass spectrometry [22, 23], which, due to the decrease in costs, may prefer to use molecular over biochemical tests. Moreover, the molecular tests are usually faster, easier and have less subjectivity in interpretation [24].

Our collaborative group has sequenced more than 60 *C. pseudotuberculosis* strains isolated from different hosts around the world, performing genomic, proteomic and clinical studies to not only to understand the pathogen but also try to find a way to control the spread of bacteria. Thus, the aim of this study was to develop a quadruplex PCR assay that would allow simultaneous detection and biovar-typing of *C. pseudotuberculosis* strains.

## Methods

### Nitrate reductase genes

Presence or absence of nitrate reductase genes were analyzed in nineteen *C. pseudotuberculosis* genomes (Table 1) in our previous work [21].

**Table 1:** *Corynebacterium pseudotuberculosis* strains with the whole genome sequenced available in the NCBI GenBank (www.ncbi.nlm.nih.gov/genbank) in 2015

Strain	Biovar	Host	Country	Genome size (MB)	Sequencing status	NCBI access	Reference
1002	Ovis	Goat	Brazil,	2.33511	Complete	NC_017300.1	(37)
C231	Ovis	Sheep	Australia	2.32821	Complete	NC_017301.1	(37)
FRC41	Ovis	Human	France	2.33791	Complete	NC_014329.1	(38)
I19	Ovis	Cow	Israel	2.33773	Complete	NC_017303.1	(39)
PAT10	Ovis	Sheep	Argentine	2.33532	Complete	NC_017305.1	(40)
42/02-A	Ovis	Sheep	Australia	2.33761	Complete	NC_017306.1	(41)
3/99–5	Ovis	Sheep	Scotland	2.33794	Complete	NC_016781.1	(41)
267	Ovis	Llama	USA	2.33763	Complete	NC_017462.1	(6)
P54B96	Ovis	Antelope	South Africa	2.33794	Complete	NC_017031.1	(42)
CIP5297	Equi	Horse	Kenya	2.32059	Complete	NC_017307.1	(43)
1/06-A	Equi	Horse	USA	2.27912	Complete	NC_017308.1	(44)
316	Equi	Horse	USA	2.31041	Complete	NC_016932.1	(45)
258	Equi	Horse	Belgium	2.36982	Complete	NC_017945.1	(46)
162	Equi	Camel	UK	2.29346	Complete	NC_018019.1	(42)
31	Equi	Buffalo	Egypt	2.38969	Complete	NC_017730.1	(47)
262	Equi	Cattle	Belgium	2.32575	Complete	NZ_CP012022.1	–
MB20	Equi	Horse	USA	2.36309	Draft	JPUV01	(48)
E19	Equi	Horse	Unknow	2.36796	Complete	NZ_CP012136.1	–
CCUG27541	Equi	Horse	Unknow	2.37942	Draft	JPJB01	(49)

### Bacterial strains and culture conditions

A total of 348 *C. pseudotuberculosis* strains, 346 field isolates [25–27], *C. pseudotuberculosis* ATCC 19410^T^ type strain, and *C. pseudotuberculosis* 1002 vaccine strain, were used in this study. These strains were obtained from the repository of the Laboratório de Genética Celular e Molecular, Instituto de Ciências Biológicas and of the Laboratório de Bacteriologia Aplicada, Escola de Veterinária of the Universidade Federal de Minas Gerais. *C. pseudotuberculosis* biovars Ovis and Equi were aerobically grown in brain heart infusion (BHI) (Acumedia Manufacturers, Baltimore, USA) agar plates at 37 °C for 48 h. The *C. pseudotuberculosis* isolates were identified by standard biochemical tests [9, 28, 29]. Nitrate reduction was assessed using nitrate broth (Merck, Billerica, USA) and further reduction beyond nitrite was tested by addition of zinc dust (Sigma-Aldrich, St Louis, USA) [28].

### Genomic DNA extraction, primers, and quadruplex PCR

Genomic DNA extraction were performed according to the previously described protocol [30]. The oligonucleotide primers used in this study are listed in Table 2. Primers used to target *16S* rRNA, *rpoB,* and *pld* genes of *C. pseudotuberculosis* were previously described [14, 31, 32]. Primers targeting the *narG* gene were designed by aligning the *narG* nucleotide sequences of *C. pseudotuberculosis* biovar Equi strains available from the whole genome sequenced strains (Table 1). Quadruplex PCR were carried out in a final volume of 50 μL, containing 20 ng of genomic DNA, 1 μM of each primer, 0.25 mM dNTPs, 1 units of *Taq* DNA polymerase (Life Technologies, Carlsbad, USA), 2 mM MgCl_2_, and 1X buffer (200 mM Tris-HCl pH 8.4, 500 mM KCl) (Life Technologies, Carlsbad, USA). Amplification was performed using the thermal cycler (PTC-100, MJ Research, Hercules, USA) as follows: the first denaturation at 95 °C for 4 min; followed by 30 cycles of denaturation at 95 °C for 30 s, annealing at 58 °C for the 30s, and extension at 72 °C for 1.5 min. The amplified products were submitted to electrophoresis in 1.0% agarose gel (*w*/*v*) in Tris-borate-EDTA (TBE) buffer (89 mM Tris Base, 89 mM Boric Acid and 2 mM EDTA pH 8.0), stained with 0.5 mg / mL ethidium bromide and visualized under UV light.

**Table 2:** List of oligonucleotide primers used in this study

Target gene	Primers	Sequence (5′ → 3′)	Amplicom size (bp)	Multiplex PCR assay	Reference
*16S rRNA* ^*a*^	Forward	ACCGCACTTTAGTGTGTGTG	816	Yes	(25)
	Reverse	TCTCTACGCCGATCTTGTAT			
*rpoB* ^*a*^	Forward	CGTATGAACATCGGCCAGGT	446	Yes	(26)
	Reverse	TCCATTTCGCCGAAGCGCTG			
*pld* ^*a*^	Forward	ATAAGCGTAAGCAGGGAGCA	203	Yes	(14)
	Reverse	ATCAGCGGTGATTGTCTTCCAGG			
*narG* ^*a*^	Forward	ACCCGTACTTGCACTCTTTC	612	Yes	Present Study
	Reverse	AGTCAGTACTTCCGCAGGTC			
*narT*	Forward	GCTGAAGCAAGTTCGTGTCT	202	No	Present Study
	Reverse	GTAACGGTCAGAGAACCATCC			
*narK*	Forward	GCTGAAGCAAGTTCGTGTCT	202	No	Present Study
	Reverse	GTAACGGTCAGAGAACCATCC			
*narG2*	Forward	CAACGTGGTACCTGGTATCTG	200	No	Present Study
	Reverse	CATAGGGAGAGCGAGAACAA			
*narH*	Forward	GATTCTACTGACCGCCATCTC	196	No	Present Study
	Reverse	ATCAGTACCTGTCATGCCTACC			
*narJ*	Forward	CGTGATGGTATAGAGGTGCTG	198	No	Present Study
	Reverse	GTTGGAAGCAGTAGGGAAGGGAG			
*narI*	Forward	CTGTATCCACACAGGTGTTCG	215	No	Present Study
	Reverse	GTATCCTACAGGCGCTGAGA			

^*a*^Primers used to quadriplex PCR assay

### Sequencing of singleplex PCR products

In order to confirm the quadruplex PCR results, ten randomly chosen isolates were tested further in singleplex PCR assays with the four *C. pseudotuberculosis*-specific primer pairs. PCR products were purified using Agencourt AMPure XP (Beckman Coulter Company, Beverly, Massachusetts, USA) according to the manufacturer’s instructions, and each product was sequenced in both directions using primers targeting the 16S rRNA, *rpoB*, *pld* and *narG* gene and the Big Dye V3.1 Terminator Kit (Applied Biosystems, USA) using an ABI 3500 DNA analyzer (Applied Biosystems, California, USA). Sequences were analyzed on the Geneious suite of molecular biology (http://www.geneious.com) with 16S rRNA (GenBank accession nos X81916, X81907, and X84255), *rpoB* (GenBank accession no. AY492239), *pld* (GenBank accession nos L16586 and L16587) and *narG* (GenBank accession no AJF93840.1) as the reference genes.

### Statistical analysis

Comparison between nitrate reduction test and quadruplex PCR was performed by McNemar’s Chi-squared test, and the agreement was calculated using the kappa statistic. Statistical analysis were performed using the packages psych [33] and epibasix [34] on R software version 3.2.3 [35].

## Results

Comparative genome analysis showed that *C. pseudotuberculosis* biovar Equi strains (258, 31, 262, MB20, E19 and CCUG27541) had *narKGHJI* gene clusters, however strains 1/06-A, 316, 162, and CIP52.97, although showing positive results in the nitrate reduction test, did not exhibit *narKGHJI* operon in their genome. On the other hand, genomic sequence analysis identified partial genes *molB, narJ, moeA,* and *moeB* in the strains 1/06-A, 162 and CIP52.97.

Since the strains 1/06-A, 316, 162, and CIP52.97 were nitrate reductase positive in biochemical test and the genes were not identified in their genomes, primers to target *narKGHJI* cluster and *narT* gene were designed (Table 2). The PCR tests (Additional file 1: Figure S1), sequencing and the optical map showed that the genes for *narKGHJI* and *narT* are present in the genomes of those strains (data not shown).

**Fig. 1:**
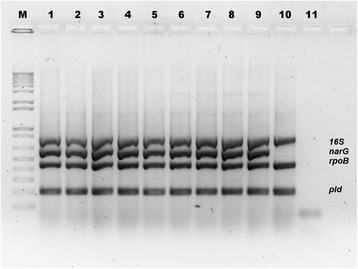
Four-primer quadruplex PCR for *C. pseudotuberculosis* species and biovar identification.Agarose gel 1.5% showing the PCR amplification of quadruplex PCR assay stained with ethidium bromide (0.5 mg / mL).L: GeneRuler DNA Ladder (Fermentas, Vilnius, Lithuania); Lanes 1–9:*C. pseudotuberculosis* biovar Equi strains C31, 258, 262, 162, 5297, 1/06A, EG-37, EG-42 and I-37; Lane 10:*C. pseudotuberculosis* biovar Ovis strain 1002

The multiplex PCR assay that targets *16S rRNA*, *rpoB* and *pld* genes [14] was improved by the inclusion of *C. pseudotuberculosis* biovar-specific primers for the *narG* gene (*narG* – Table 2), in a novel quadruplex PCR assay (Fig. 1). The assessment of our quadruplex PCR assay was performed in a double-blind fashion. The results of the quadruplex PCR of the 348 previously well-characterized strains of *C. pseudotuberculosis* from different hosts (goats, sheep, horse, cattle, buffalo, llamas and humans) [21, 25–27] were compared to the results of nitrate reductase identification by biochemical test, and are shown in Table 3. The McNemar’s Chi-squared test used to compare the two methods employed for *C. pseudotuberculosis* biovar identification showed no significant difference (*P* = 0.75), with an odds ratio of 1 (95% CI for the odds ratio: 0.16–6.14) between the quadruplex PCR and the nitrate biochemical test. Concordant results were observed for 97.13% (338/348) of the strains (Table 3), and the kappa statistic value was 0.94 [95% CI (0.90–0.98)], denoting excellent concordance between biochemical and molecular tests for nitrate reductase identification. The limit of detection of the new quadruplex PCR was 200 ng of DNA from *C. pseudotuberculosis* biovar Equi, which corresponds to approximately 100 bacteria.

**Table 3:** Comparison of biochemical test and a multiplex PCR assay employed for *Corynebacterium pseudotuberculosis* biovar identification

Multiplex PCR assay	Biochemical test		TOTAL
	Nitrate positive	Nitrate negative	
Nitrate positive	133	5	138
Nitrate negative	5	205	210
Total	138	210	348

McNemar’s Chi-squared test = P = 0.75, Odds Ratio: 1 (95% CI for the odds ratio: 0.16–6.14)

Kappa coefficient = 0.94 (95% CI: 0.91–0.98)

## Discussion

Previously, identification of *C. pseudotuberculosis* biovars was only possible only through the established procedures that included isolation and identification of the agent using biochemical tests such as the nitrate reduction test, which separates the nitrate-positive biovar Equi from nitrate negative biovar Ovis strains [5]. Herein, we developed, by the addition of a new oligonucleotide primer pair targeting the *nar*G gene to the former multiplex PCR assay [14], a robust new assay for identification of *C. pseudotuberculosis* at species and biovar levels.

The comparative genome analysis showed in *C. pseudotuberculosis* biovar Equi strains (258, 31, 262, MB20, E19 and CCUG27541) *narKGHJI* gene clusters that participate via the respiratory anaerobic process of the nitrate reduction similar to *Escherichia coli* [21, 36]. The *C. pseudotuberculosis narKGHJI* gene cluster showed significant similarity with the protein sequences found in other Actinomycetes, such as *C. diphtheriae*, *C. glutamicum,* and *Mycobacterium tuberculosis.* All *C. pseudotuberculosis* biovar Ovis strains do not present any gene of the *narKGHJI* operon in their genomes [21].

**Fig. 2:**
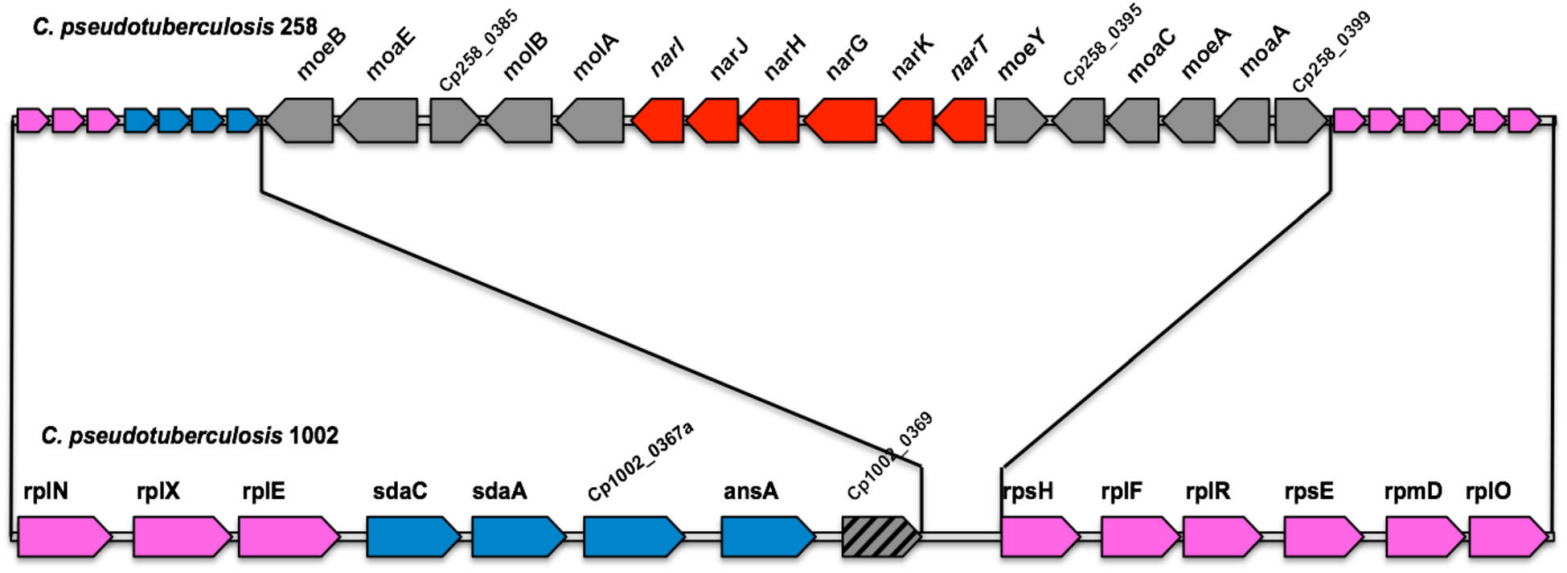
Nitrate *locus* from *C. pseudotuberculosis* biovar Equi.This *locus* contains: the genes encoding the molybdopterin *moeB, moaE, molB, molA, moeY, moaC, moeA* and *moaA* and the genes encoding the nitrate reductase *narK*, *narG*, *narH*, *narJ*, *narI*. Insertion show between *ansA* and *rpsH* genes is lacking in nitrate negative *C. pseudotuberculosis* biovar Ovis strains. Arrows represent open reading frames and their orientations. Blue and pink: common genes shared between *C. pseudotuberculosis* biovar Ovis and biovar Equi strains. Pink: ribosomal proteins. Hatched: additional or different genes. Red: *narKGHJI* operon and grey: genes encoding the molybdopterin *moeB, moaE, molB, molA, moeY, moaC, moeA* and *moaA*

The nitrate *locus* in *C. pseudotuberculosis* is composed of the *narKGHJI* operon and by a cluster of genes encoding the molybdopterin *moeB, moaE, molB, molA, moeY, moaC, moeA,* and *moaA* (Fig. 2) [21]*.* Molybdopterin is a cofactor that is indispensable for the activity of nitrate reductase. In the *narGHI* complex, the *narG* gene is a member of a superfamily of enzymes that use a Molybdopterin-guanine-dinucleotide (Mo-bisMGD) cofactor (bisMGD) for their catalytic activity.

Our results showed that among the 348 *C. pseudotuberculosis* tested, only 10 strains (2.87%) showed differences between the biovar classification provided by molecular and biochemical tests (Table 3), which was observed even after repeating the assays. Furthermore, the kappa coefficient, which is a robust statistic that measures inter-rater agreement for qualitative items, confirms that regardless of the technique used for biovar classification the results obtained were very similar. It is also important to consider that kappa values range from −1 to +1, where 1 represents a perfect agreement between the raters, and 0.81 to 1.00 represents almost perfect agreement, such as the observed in our data (0.94) [28, 37]. Discordance between both phenotypic and genotypic methods can be explained by the effect of environmental factors on gene expression [29, 38] amino-acid substitution, the genetic background of the strain (that can vary according to geographical locations) and mutations. Considering discordances due to mutations genotypic tests have proven to be more reliable and sensitive as diagnostic tool than phenotypic tests [30–32, 39–42]. Others studies also showed discordant results between genotypic versus phenotypic methods [30–32, 39–41, 43, 44].

To our acknowledgement, this is the first molecular approach able to clearly differentiate between *C. pseudotuberculosis* biovar Ovis and Equi, although different restriction patterns, ribotypes and ERIC-PCR clustering pattern have been associated to biovars [26, 36, 45–47]. The advantages of multiplex PCR assay over biochemical tests are the speed, performance and reproducibility, and the ability to test large numbers of isolates simultaneously [26, 45, 46]. Identification is based upon the number and sizes of four products amplified by PCR. Moreover, the use of molecular techniques reduces the manipulation of viable bacteria in the laboratory and consequently the risk of accidental infection, as *C. pseudotuberculosis* can eventually be a zoonotic agent [2]. Moreover, this new diagnostic tool, the quadruplex PCR assay for identification and biotyping of *C. pseudotuberculosis* follows the new trends on clinical microbiology laboratory that is currently incorporating more molecular biology tools in its routine [23, 48]. In addition, despite not having been tested in the present study, due to its analytical sensitivity of 100 bacteria. |It is likely that this quadruplex PCR can also be applied to direct testing from clinical samples, as it has been done successfully for the three-primer (*16S* rRNA, *rpoB,* and *pld*) multiplex PCR [14].

The quadruplex PCR proposed in this study facilitates and deepens the level of identification of *C. pseudotuberculosis* strains at clinical microbiology laboratory, and thereby improves the diagnosis of infection by providing more information for decision making. These results are especially significant considering that *C. pseudotuberculosis* infects a wide range of hosts and produce different clinical manifestations. Furthermore, it was recently suggested that *C. pseudotuberculosis* biovars have differences at the molecular phylogenetic level, indicating an anagenesis process within the species [37, 49]. The evolutionary analysis of conserved genes (*rpoB, gapA, fusA,* and *rsmE*) suggesting a gradual anagenesis of *C. pseudotuberculosis* in that study [49] substantially increases the importance of a molecular technique capable of efficiently separating the biovars Ovis and Equi. In fact, a pan-genome analysis of fifteen *C. pseudotuberculosis* strains showed a significant number of genes not shared by both biovars, including remarkable differences in the 16 detected pathogenicity islands [50].

In this study, it was also observed that some *C. pseudotuberculosis* strains (1/06-A, 316, 162 and CIP52.97) were able to reduce nitrate when tested by the biochemical approach and were also positive in the quadruplex PCR assay, albeit did not show in their genomes genes associated with nitrate reduction. The genomic analysis of nitrate *locus* identified that partial genes encoding the molybdopterin and *narKGHJI* operon of these strains was absent [21]. These results may have been due to low overall coverage, poor capture efficiency of certain regions, genomic regions that were previously not assembled or poorly assembled, including unambiguously aligning repetitive regions, such as transposons, and difficulty in unambiguously aligning repetitive regions [41, 51]. Then, after resequencing of the *narKGHJI* operon region and optical mapping of these strains, it was observed that these strains have the nitrate *locus* in their genome and corrections on their information on GenBank are under way.

## Conclusions

A novel quadruplex PCR assay for *C. pseudotuberculosis* species and biovar identification was developed. The nitrate reductase gene *narG* was included in the assay along with the *16S, rpoB* and *pld* genes to improve the diagnosis of the multiplex PCR at biovar level. There was a significant concordance between the biovar classification provided by the molecular and biochemical test. The ability of the expanded quadruplex PCR assay to discriminate between *C. pseudotuberculosis* biovar Ovis and Equi strains enhances its value.

## Additional file

Additional file 1:
**Figure S1.** A and B) 1% agarose gel containing the result of PCR performed for molecular confirmation of the amplicom ***narKGHJI*** operon and *narT* gene of C31, 258, 162, 5297 and 106/A strains. (DOCX 116 kb)

## Abbreviations

CLA: Caseous Lymphadenitis
Mo-bisMGD: Molybdopterin-guanine-dinucleotide
NAR: Nitrate Reductase
OSD: Oedematous Skin Disease
