# Supplementary material for: Quadruplex PCR assay for identification of Corynebacterium pseudotuberculosis differentiating biovar Ovis and Equi
Source: BMC Vet Res. 2017 Sep 25;13:290. doi: 10.1186/s12917-017-1210-5 (PMC5613524; doi:10.1186/s12917-017-1210-5)
Supplement: Additional file 1: — Figure S1. A and B) 1% agarose gel containing the result of PCR performed for molecular confirmation of the amplicom narKGHJI operon and narT gene of C31, 258, 162, 5297 and 106/A strains. (DOCX 116 kb) [file 12917_2017_1210_MOESM1_ESM.docx]

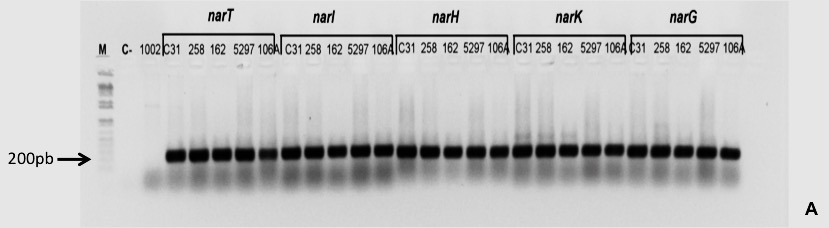

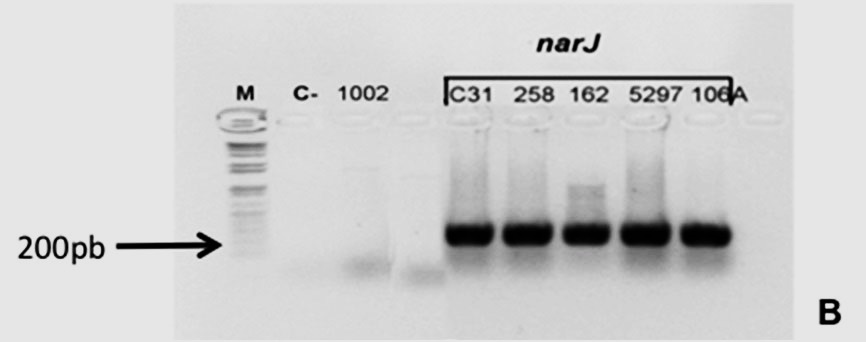


Fig. S1. A and B) 1% agarose gel containing the result of PCR performed for molecular confirmation of the amplicom *narKGHJI* operon of C31, 258, 162, 5297 and 106/A strains.
